# A Preliminary Study on the Ability of the Trypsin-Like Peptidase Activity Assay Kit to Detect Periodontitis

**DOI:** 10.3390/dj8030098

**Published:** 2020-09-01

**Authors:** Masanori Iwasaki, Michihiko Usui, Wataru Ariyoshi, Keisuke Nakashima, Yoshie Nagai-Yoshioka, Maki Inoue, Tatsuji Nishihara

**Affiliations:** 1 Tokyo Metropolitan Institute of Gerontology, 35-2 Sakae-cho, Itabashi-Ku, Tokyo 173-0015, Japan; 2 Division of Periodontology, Kyushu Dental University, Kitakyushu 803-8580, Japan; r12usui@fa.kyu-dent.ac.jp (M.U.); nakashimak@kyu-dent.ac.jp (K.N.); 3 Division of Infections and Molecular Biology, Kyushu Dental University, Kitakyushu 803-8580, Japan; arikichi@kyu-dent.ac.jp (W.A.); r16yoshioka@fa.kyu-dent.ac.jp (Y.N.-Y.); r20inoue@fa.kyu-dent.ac.jp (M.I.); tatsujin@kyu-dent.ac.jp (T.N.)

**Keywords:** epidemiology, oral health, periodontal diseases, population surveillance, receiver operating characteristics curve

## Abstract

This study aimed to explore whether the Trypsin-Like Peptidase Activity Assay Kit (TLP-AA-Kit), which measures the activity of N-benzoyl-dl-arginine peptidase (trypsin-like peptidase), can be used as a reliable tool for periodontitis detection in population-based surveillance. In total, 105 individuals underwent a full-mouth periodontal examination and provided tongue swabs as specimens for further analyses. The results of the TLP-AA-Kit were scored between 1 and 5; higher scores indicated higher trypsin concentrations. Receiver operating characteristic analyses were used to evaluate the predictive validity of the TLP-AA-Kit, where the periodontitis case definition provided by the Centers for Disease Control/American Academy of Periodontology served as the reference. Severe and moderate periodontitis were identified in 4.8% and 16.2% of the study population, respectively. The TLP-AA-Kit showed high diagnostic accuracy for severe periodontitis, with an area under the curve of 0.93 (95% confidence interval = 0.88–0.99). However, the diagnostic accuracy of the TLP-AA-Kit for moderate/severe periodontitis was not reliable. While further studies are necessary to validate our results, the results provided herein highlight the potential of the TLP-AA-Kit as a useful tool for the detection of periodontitis, particularly in severe cases, for population-based surveillance.

## 1. Introduction

Periodontitis is a common disease worldwide. Based on results obtained from the Survey of Dental Diseases in Japan 2016 (SDD 2016), 51% of adults aged ≥ 30 years exhibited periodontitis [1]. Similarly, a study based on the National Health and Nutrition Examination Survey 2009 to 2012 reported that the periodontitis prevalence was 46% among adults aged ≥ 30 years in the United States [2]. This disease has major adverse effects on general health, including its contribution to the development of diabetes and cardiovascular disease, as well as on an affected individual’s overall quality of life [3,4,5,6,7,8,9,10], and is associated with increased healthcare costs among the Japanese population [11]. In Japan, despite the high prevalence of periodontitis, only a small percentage of the population receives regular dental check-ups. In fact, according to a recent survey conducted by the Japan Dental Association [12], only approximately 30% of individuals surveyed reported that they visit the dentist regularly, demonstrating that many individuals are unaware of their periodontal health status. Moreover, periodontitis surveillance at the community level or in the workplace is not commonly performed in Japan [13,14]. Most cases of periodontitis are treatable [15]. Currently, the standard and preferred approach for periodontitis surveillance is through clinical examinations, with a full-mouth periodontal examination (FMPE) considered the gold standard and a partial-mouth periodontal examination (PMPE) performed when an FMPE is not available; however, a PMPE is reported to have low accuracy for surveillance and research [16]. Meanwhile, clinical periodontal examination requires manpower (trained examiner), equipment, funds, and time. In addition, the clinical periodontal examination may put individuals at a risk of bacteremia [17]. These factors restrict periodontitis surveillance at the population level. Thus, a more readily applied and less invasive surveillance method is required.

Currently, several kits for the detection of periodontitis are commercially available in Japan, including BANAPERIO (Hakusui Trading Co., Ltd., Osaka, Japan), Perioscreen (Sunstar Inc., Osaka, Japan), SillHa (ARKRAY Inc., Kyoto, Japan), and the Salivary Multi Test (Lion Dental Products Co., Ltd., Tokyo, Japan). BANAPERIO measures the activity of N-benzoyl-dl-arginine peptidase (trypsin-like peptidase), specifically produced by certain strains of *Porphyromonas gingivalis*, *Tannerella forsythia*, *Treponema denticola*, and *Capnocytophaga* [18,19], within subgingival plaque samples, ideally collected from the most diseased sites [20]. However, this platform requires the implementation of specific techniques that can be time-consuming, which may not be appropriate for population surveillance settings. Alternatively, Perioscreen detects periodontitis through immunological detection of human hemoglobin in saliva [21]. Diagnostic accuracy, including sensitivity and specificity for diagnosing periodontitis based on PMPE and not FMPE data, was assessed in a community-based population [21]. Lastly, the SillHa and Salivary Multi Test kits measure blood, leukocytes, and protein in saliva [22,23]. However, no information is available regarding the quantification measures for the diagnostic accuracy of either of these kits.

Recently, a new kit has been developed that measures the activity of trypsin-like peptidase. This Trypsin-Like Peptidase Activity Assay Kit (TLP-AA-Kit; commercially available as *ADCHECK*^®^ [ADTEC Co., Ltd., Usa, Japan]) uses tongue swab specimens. Hence, sample collection is completed within seconds using this less invasive platform that does not require trained professionals. The TLP-AA-Kit, therefore, has the potential to become a useful tool for periodontitis surveillance, but its diagnostic performance must first be validated in a real-world setting. The aim of the current study was to explore whether this kit is effective for detecting periodontitis in Japanese adults under the null hypothesis that the diagnostic accuracy of the test (area under the curve [AUC]) would be 0.50.

## 2. Materials and Methods

### 2.1. Study Design, Setting, and Participants

The TLP-AA-Kit was evaluated against a periodontal diagnosis based on an FMPE. The study population comprised a cohort of individuals who worked at Yaskawa Electric Corporation, Japan. Inclusion criteria were as follows: at least 20 years of age, able to read and understand Japanese, presence of ≥1 tooth, and willingness to undergo periodontal examination. Exclusion criterion was pre-diagnosed severe or terminal disease, such as advanced-stage cancer or advanced heart failure. All study participants provided written informed consent prior to enrollment. This study was conducted in full accordance with the ethical principles of the Declaration of Helsinki and was approved by the Ethics Committee of Kyushu Dental University (Approval number: 17-47, Date of approval: 30 March 2018). Furthermore, the Strengthening the Reporting of Observational studies in Epidemiology (STROBE) statement was followed.

Sample size calculation was performed using R version 3.3.3 (R Foundation for Statistical Computing, https://www.r-project.org). Considering the prevalence of periodontitis observed in the SDD 2016 (49.4%) (https://www.mhlw.go.jp/toukei/list/dl/62-28-02.pdf), the sample size was estimated as 105 individuals based on an AUC = 0.70, corresponding to a one-sided alpha = 0.05, power = 95%, and attrition rate = 5%.

### 2.2. Data Collection

#### 2.2.1. Questionnaire Administration

Age, sex, alcohol consumption, smoking status, exercise habits, sleep quality, and diabetes status were collected through self-administered questionnaires. Details of the frequency and amount of alcohol consumed were required; according to the second phase of Healthy Japan 21 and related studies [24,25], high-risk drinking was defined as a response of “every day” or “sometimes” to the question “how often do you drink: every day; sometimes; or rarely,” and an answer of “2 drinks or more” to the question “how many drinks containing alcohol do you have on a typical day when drinking: <1 drink; 1 drink; 2 drinks; and ≥3 drinks.” Smoking status was dichotomized into current smoker or not. Habitual exercise was defined as an answer of “yes” to the question “Do you engage in exercise with ≥30 min per session, ≥2 times per week and prolonged duration for ≥1 year?” Sleep quality was defined as poor by an answer of “no” to the question “Do you take enough rest by sleeping?”

Furthermore, health status and behavior prior to the TLP-AA-Kit examination (i.e., antibiotic usage within a month; tooth brushing within an hour; tongue cleaning within an hour; mouth rinse usage within an hour; having eaten within an hour; and smoking within an hour) were collected via questionnaire.

#### 2.2.2. TLP-AA-Kit

TLP-AA-Kit was used before oral health examination as per the manufacturer’s instructions. Tongue swabs were collected from participants and placed in the extraction buffer and stirred. Each swab was then pressed onto a test plate disk for 5 s and allowed to react for 10 min at 20–30 °C. The test plate disks contained Nα-benzoyl dl-arginine β-naphthylamide hydrochloride as a matrix. If trypsin-like peptidase was present in the swab, the enzymatic activity of relevant enzyme in the swab was expected to cause disintegration of the matrix and a subsequent release of β-naphthylamide.

After 10 min, one drop of a 4-(dimethylamino)cinnamaldehyde color developer was placed onto the test plate; β-naphthylamide if released reacted to produce a pink-colored chemical compound. Three minutes after application of the color developer, the intensity of the matrix disk color was assessed by visual inspection using the score interpretation samples. The matrix disk was ranked in increments of 0.5 units (range 1–5) for the intensity of the disk color, with a strong pink color (i.e., high score) indicating intense trypsin-like peptidase activity. The scoring system was as follows: 1 was equivalent to ≥10 units/mL of trypsin, 2 was equivalent to ≥25 units/mL, 3 was equivalent to ≥100 units/mL, 4 was equivalent to ≥500 units/mL, and 5 was equivalent to ≥5000 units/mL. One unit is defined as the amount of enzyme required to produce a ΔA253 of 0.001 per minute with Nα-benzoyl-l-arginine ethyl ester as the substrate, at pH 7.6 and 25 °C.

The kit test was performed by one examiner blinded to the periodontal status of the participants.

#### 2.2.3. Oral Health Examinations

Three trained dentists (inter-examiner kappa values of >0.8) blinded to the scores of the TLP-AA-Kit assessed the probing pocket depth (PPD), gingival recession, and bleeding on probing (BOP) at six sites (mesio-buccal, mid-buccal, disto-buccal, mesio-lingual, mid-lingual, and disto-lingual) on every tooth, excluding the third molars, using a graduated periodontal probe (Williams COLORVUE^®^ Probe, Hu-Friedy Mfg. Co., LLC., Frankfurt, Germany) and a mouth mirror (HD MIRRORS, Hu-Friedy, Chicago, IL, USA). Clinical attachment loss (CAL) was calculated using the PPD and gingival recession [26,27].

For validation of the TLP-AA-Kit, the periodontitis case definition provided by the Centers for Disease Control/American Academy of Periodontology (CDC/AAP) [28] served as the gold standard for predictive validity. Following the CDC/AAP definition, the participants were classified into four groups: severe periodontitis: having ≥2 interproximal sites with a CAL of ≥6 mm (not on same tooth) and ≥1 interproximal site with a PPD of ≥5 mm; moderate periodontitis: having ≥2 interproximal sites with a CAL of ≥4 mm (not on same tooth), or ≥2 interproximal sites with a PPD of ≥5 mm (not on same tooth); mild periodontitis: having ≥2 interproximal sites with a CAL of ≥3 mm (not on same tooth) and ≥2 interproximal site with a PPD of ≥4 mm (not on same tooth) or one interproximal site with a PPD of ≥5 mm, or no periodontitis: the absence of mild, moderate or severe periodontitis.

Denture use, presence of dental plaque, and tongue-coating status were also assessed during the examination. According to a previous study [29], the presence of dental plaque was measured from three indicator teeth on one surface each as follows: the buccal surface on the most posterior tooth on the upper right side, the lingual surface on the most posterior tooth on the lower left side, and the buccal surface on the lower left canine. The presence of dental plaque was categorized into three categories: no visible plaque (assigned value 0), visible plaque on the gingival margins only (value 1), or visible plaque on gingival margins and elsewhere (value 2). The highest value for any of the indicator teeth was recorded. Tongue-coating status was assessed using the tongue-coating index (TCI) [30]. TCI quantifies the degree and extent of tongue coating on a 0%–100% scale, where higher values indicate higher degree and extent of tongue coating.

#### 2.2.4. Data Collection of Other Characteristics

Height and weight were measured to calculate body mass index (BMI). Obesity was defined as a BMI ≥ 25 kg/m^2^.

Blood samples were drawn for the measurement of blood glucose and glycated hemoglobin A1c (HbA1c) levels. Diabetes was defined as self-reported physicians’ diagnosis, and/or self-reported use of insulin or other glucose-lowering drugs, and/or fasting blood glucose level of ≥126 mg/dL, and/or random blood glucose level of ≥200 mg/dL, and/or HbA1c of ≥6.5%.

### 2.3. Statistical Analyses

Statistical analyses were carried out using Stata version 15.1 (Stata Corporation LP, College Station, TX, USA), with the level of significance (two-tailed) set at 0.05. The Standards for Reporting Diagnostic Accuracy (STARD) 2015 guidelines [31] were followed for reporting diagnostic accuracy studies.

Descriptive statistics were performed to characterize the study population and compare the groups according to the presence and severity of periodontitis. Analysis of variance, the Kruskal–Wallis test, or Fisher’s exact test was used as appropriate. If the overall test was significant, post-hoc comparisons were performed.

The TLP-AA-Kit scores between the groups according to the health status and behavior prior to the examination, as well as among the groups, according to the presence and severity of periodontitis were compared using the Kruskal–Wallis test. If the overall test was significant, post-hoc comparisons were performed.

The associations between TLP-AA-Kit score and periodontal parameters, including average PPD, average CAL, and BOP, were examined using Spearman’s rank correlation.

The sensitivity and specificity of the TLP-AA-Kit were calculated using the CDC/AAP definition as the reference standard. Sensitivity and specificity values were considered high if values were ≥80%, moderate if 80% > values ≥ 60%, and low if values were <60% [32]. Two models were tested: (i) the first model predicted “moderate + severe periodontitis” versus “no + mild periodontitis” and (ii) the second model predicted “severe periodontitis” versus “no + mild + moderate periodontitis.” For each model, parameter estimates were used to calculate the predicted probabilities of having periodontitis for each participant, and values were plotted against observed periodontitis cases to generate receiver operating characteristics (ROC) curves. The predictive validity of the models was assessed by calculating the AUC. The accuracy determined using the AUC was interpreted as high if AUC > 0.8, useful if 0.8 ≥ AUC > 0.7, and low if AUC ≤ 0.7 [33]. The optimal cut-off thresholds to identify individuals with periodontitis were determined using the highest Youden’s index (sensitivity + specificity − 1). A sum of sensitivity and specificity > 120% is considered to have good validity [34].

The performance of the TLP-AA-Kit as a periodontitis surveillance tool was also assessed based on sensitivity, specificity, and ROC among subgroups without a smoking habit, obesity, or diabetes.

## 3. Results

### 3.1. Study Population Characteristics

The study population comprised 105 adults who met the inclusion criteria and provided complete data. Based on the CDC/AAP definition of periodontitis, 4.8% of the study population (5/105 participants) was categorized as having “severe” periodontitis, 16.2% (17/105 participants) as having “moderate” periodontitis, 10.5% (11/105 participants) as having “mild” periodontitis, and 68.6% (72/105 participants) as having “no” periodontitis.

Table 1 shows the comparison of variables that describe the study population’s characteristics among four groups according to the periodontitis classification (severe, moderate, mild, and no). Variables that exhibited significant differences among groups were average PPD and CAL, BOP, age, obesity, and diabetes. Specifically, the group without periodontitis had lower BOP and average PPD and CAL, were younger, and were less likely to be obese or diabetic.

### 3.2. TLP-AA-Kit Score

The TLP-AA-Kit score was also compared between the groups according to the selected behavior prior to examination (i.e., antibiotic usage within a month, tooth brushing within an hour, tongue cleaning within an hour, mouth rinse usage within an hour, having eaten within an hour, and smoking within an hour); however, no significant differences were observed for any of the results (Table 2).

### 3.3. Association between TLP-AA-Kit Score and Periodontitis

**Figure 1:**
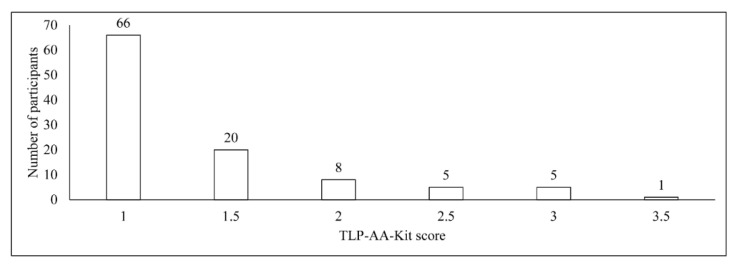
Frequency table of participants according to the Trypsin-Like Peptidase Activity Assay Kit (TLP-AA-Kit) score.

The frequencies of participants diagnosed via TLP-AA-Kit score are presented in Figure 1. The median (interquartile range [IQR]) of the TLP-AA-Kit scores among study participants was 1.0 (1.0–1.5).

Table 3 shows the correlation between TLP-AA-Kit score and periodontal parameters. TLP-AA-Kit score was significantly positively correlated with average PPD (Spearman’s rank correlation coefficient [ρ] = 0.224, *p* = 0.022) and average CAL (ρ = 235, *p* = 0.016), whereas the correlation between TLP-AA-Kit score and BOP was only trending toward significance (ρ = 0.164, *p* = 0.095).

**Figure 2:**
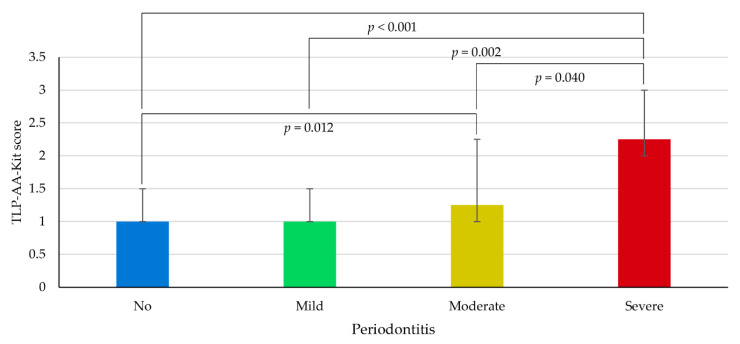
TLP-AA-Kit scores according to periodontitis classification.

The TLP-AA-Kit scores according to periodontitis classification are presented in Figure 2. The median (IQR) values of TLP-AA-Kit scores among study participants with severe, moderate, mild, and no periodontitis were 2.25 (2.0–3.0), 1.25 (1.0–2.25), 1.0 (1.0–1.5), and 1.0 (1.0–1.5), respectively. The TLP-AA-Kit score in the severe periodontitis group was significantly higher than that of the other three groups (post-hoc comparisons; *p* < 0.05).

### 3.4. ROC Analysis

**Figure 3:**
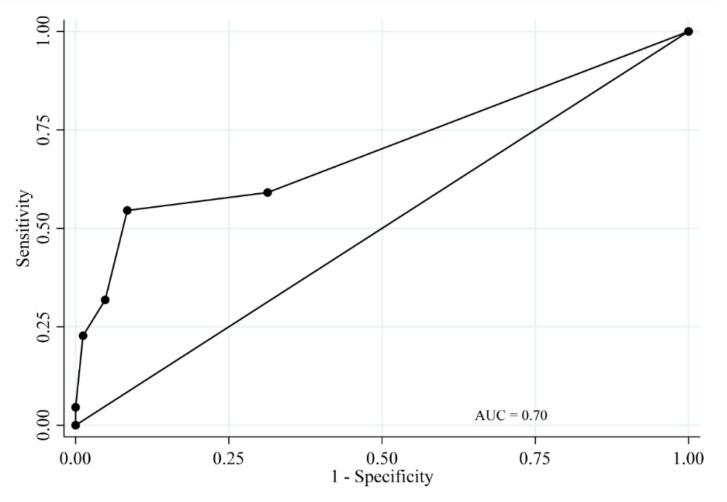
Receiver operating characteristic curve for the TLP-AA-Kit score for moderate/severe periodontitis.

**Figure 4:**
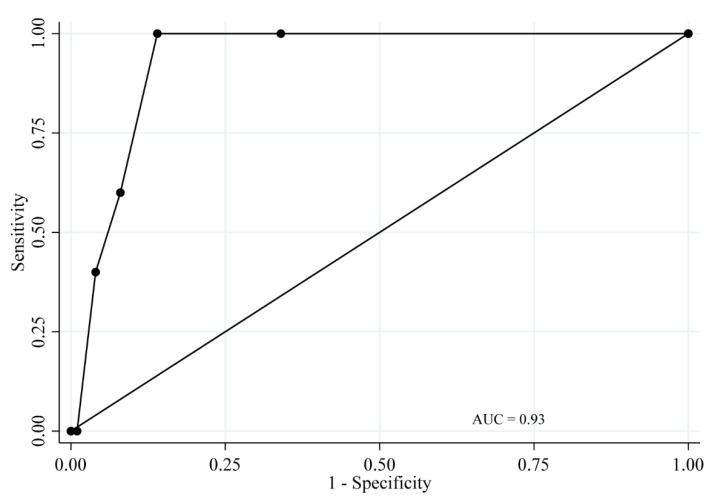
Receiver operating characteristic curve for the TLP-AA-Kit score for severe periodontitis.

Sensitivity, specificity, and correct classification, as well as positive and negative likelihood ratios of the TLP-AA-Kit score for periodontitis cases, are presented in Table 4. In addition, the ROC curves of the TLP-AA-Kit scores are presented in Figure 3 (for detecting participants with moderate periodontitis and severe periodontitis [moderate/severe periodontitis]) and Figure 4 (for detecting participants with severe periodontitis). The diagnostic accuracy of the TLP-AA-Kit score for moderate/severe periodontitis was not high (AUC = 0.70, 95% confidence interval [CI] = 0.57–0.84). However, the diagnostic accuracy of the TLP-AA-Kit score for severe periodontitis was high, with an AUC of 0.93 (95% CI = 0.88–0.99). The cut-off score to best differentiate individuals with severe periodontitis was ≥2 points, with a sensitivity of 100% and specificity of 86.0% (Table 4). Both sensitivity and specificity were considered high, and the sum of sensitivity and specificity (186.0%) indicated strong validity for this method.

Furthermore, subgroup analyses demonstrated that the TLP-AA-Kit had good diagnostic performance for severe periodontitis, with an AUC of 0.95 (95% CI = 0.89–1.00) in participants without a smoking habit, obesity, or diabetes (n = 73; Table S1 and Figure S1).

## 4. Discussion

The key findings in the current study were as follows: (i) severe periodontitis was diagnosed in 4.8% of the current study population. (ii) The TLP-AA-Kit can be used to identify individuals with severe periodontitis. (iii) The diagnostic capacity of the TLP-AA-Kit was independent of potential confounders such as smoking, obesity, or diabetes. Hence, results obtained from the TLP-AA-Kit may prompt an individual who does not regularly visit the dentist to seek treatment for periodontitis.

Previous studies have shown that periodontitis, particularly in severe cases, is associated with serious adverse health events, including cardiovascular disease and death [9,10]. Epidemiological surveillance of severe periodontitis is thus important [33]. However, the surveillance of periodontitis at the population level remains challenging. A useful, reliable, and user-friendly tool for such surveillance is needed. Moreover, compliance with continuous periodontal maintenance is the key to prevention of tooth loss [35]. Therefore, future studies investigating whether application of the TLP-AA-Kit effectively monitors periodontal health in such a way as to motivate patients to adhere to periodontal maintenance therapy would be of interest.

The current designed score (range 1–5) of the TLP-AA-Kit may not be appropriate for the detection of moderate periodontitis. In this study population, no TLP-AA-Kit scores were determined to be ≥4, indicating that the scoring should be re-designed for lower levels of trypsin concentration. Further studies are necessary to investigate the application of a newly designed scoring system that aims to detect mild and moderate periodontitis more accurately. As periodontitis, especially in mild cases, often has no or very few subjective symptoms, developing a diagnostic marker that can be used to detect the early stage of this disease is important to inform individuals of their periodontal health status.

Aside from the TLP-AA-Kit, various screening and surveillance tools for periodontitis are available that have the potential to substitute for clinical periodontal examinations. For instance, the self-report questionnaire is the most widely proposed strategy for periodontitis surveillance. According to a recent systematic review [36], sensitivity and specificity of a self-report questionnaire ranged from 4% to 93%, and from 58% to 94%, respectively. Furthermore, it is a low-cost and low-resource option. However, this tool is inherently biased by the person’s state of mind at the time they complete the questionnaire [37]. The results of self-reports are also affected by a person’s cognitive state. Alternatively, several biomarkers in saliva, gingival crevicular fluid (GCF), and blood have been used for periodontitis surveillance/diagnosis. Specifically, quantifying saliva hemoglobin for the surveillance of periodontitis has a sensitivity of 72%–76% and specificity of 52%–76% [21,38]. Moreover, according to a recent systematic review [39], macrophage inflammatory protein-1 alpha (MIP-1α), interleukin-1 beta (IL-1β), interleukin-6 (IL-6), and matrix metalloproteinase-8 (MMP-8) were identified as potentially useful salivary biomarkers. Sensitivity and specificity ranges were 66%–95% and 68%–97% for MIP-1α, 53%–88% and 52%–100% for IL-1β, 53%–80% and 48%–87% for IL-6, and 65%–87% and 48%–87% for MMP-8, respectively. Additionally, metabolic profiling of saliva (combination of cadaverine, 5-oxoproline, and histidine) identified individuals, according to the CDC/AAP definition, with moderate/severe periodontitis with an AUC of 0.88 [40]. Moreover, a meta-analysis was recently performed for four biomarkers in GCF: MMP-8, elastase, cathepsin, and trypsin [41]. The median sensitivity and specificity were 77% and 92% for MMP-8, 75% and 81% for elastase, 73% and 67% for cathepsin, and 71% and 66% for trypsin, respectively. A blood IgG antibody titer test against *P. gingivalis* identified the individuals who had periodontal lesions (PPD ≥ 4 mm) with a sensitivity of 77%, specificity of 59% and an AUC of 0.71 [42]. However, as different definitions for periodontitis were used across studies, it is difficult to perform direct comparisons regarding the effectiveness of periodontitis detection methods among these studies. Nevertheless, the validity of the TLP-AA-Kit for surveillance of periodontitis is comparable to that of methods presented in other studies. Regarding sample collection, compared to GCF, the TLP-AA-Kit is less technically sensitive, and compared to blood biomarkers, the TLP-AA-Kit is less invasive and costly. Furthermore, sample collection for the TLP-AA-Kit requires only a few seconds, and results are obtained within 15 min, which is essential for its application in mass surveillance.

Assessment of the trypsin-like peptidase activity in subgingival plaques as a potential diagnostic marker for periodontitis was introduced in the 1980s [43]. Although it has potential usefulness for population surveillance, to date, it has not been broadly used, as the information obtained is highly dependent on the sampling technique and is relevant to the site sampled, and hence, may not be representative of the microflora of the entire dentition [44]. These limitations may interfere with the use of this test in population-based surveillance.

Importantly, the results of the TLP-AA-Kit were not associated with the individual’s behavior, such as tooth brushing or smoking, prior to examination, indicating that no strict sampling conditions are required. However, the median TLP-AA-Kit score was 1.25 in the group with mouth rinse usage within an hour, and 1 in the group without its usage, which did not account for a statistically significant difference between the groups (*p* = 0.548; Table 2). Considering that only six participants (5.7%) reported the use of mouth rinse within an hour of testing, the strength and/or direction of the association may change if the sample size were to be increased, or alternatively, the observed higher median score in the mouth-rinse group may have been caused by chance. Furthermore, the results of this study were strengthened by comparing the diagnostic accuracy of the TLP-AA-Kit against an FMPE performed by trained and calibrated examiners, as well as by applying the CDC/AAP definition for periodontitis [28], which is the most frequently used definition and is considered the most appropriate in epidemiological settings [45]. However, the CDC/AAP case definition does not take into account BOP, which could indicate active inflammation. In our results, we observed a significant association between periodontitis severity and BOP (*p* = 0.029; Table 1). However, the correlation between TLP-AA-Kit score and BOP was only trending toward significance (*p* = 0.095; Table 3). Hence, the combined use of the TLP-AA-Kit and other kits related to BOP could provide additional information regarding the role of active inflammation in periodontal disease status. Further studies are therefore needed to investigate these combinatory methods.

A new classification scheme for periodontal and peri-implant diseases and conditions was introduced in the workshop of AAP and European Federation of Periodontology (EFP) [46]. Periodontitis staging based on this AAP/EFP classification includes the assessment of interdental CAL (or radiographic bone loss, if CAL is not available), tooth loss due to periodontitis, and complexity factors, such as PPD, furcation involvement, and tooth mobility. However, because the current study protocol was designed before the publication of this AAP/EFP classification, it was not applied here for periodontitis staging due to the absence of information pertaining to the causes of tooth loss, furcation involvement, and tooth mobility. Future work will seek to assess the diagnostic accuracy of the TLP-AA-Kit for periodontitis based on the new AAP/EFP classification.

Several limitations were noted in this study. First, the number of severe periodontitis cases observed in this study was small (only five participants). As the prevalence of periodontitis based on the CDC/AAP definition among the general population in Japan is not available, the sample size for this study was calculated based on the observed prevalence of periodontitis defined by PMPE in SDD 2016 [1]. Hence, in future studies, the sample size calculation should be based on the observed prevalence of periodontitis in the study. Overall, it should be noted that no definitive conclusion was drawn owing to the small number of cases of severe periodontitis. Therefore, the current study is preliminary in nature, and future large-scale studies are required to verify the findings presented herein. Second, the study population may not be representative of the general Japanese population since selection bias may have occurred as the periodontal examination was performed on a voluntary basis. When the periodontitis definition used in the SDD 2016 is applied to the current study population, periodontitis prevalence is 47.6%. Although the observed prevalence of periodontitis in this study is comparable to that reported in SDD 2016, future studies are needed to verify the external validity of the present findings.

## 5. Conclusions

Despite the limitations of this study, we demonstrate that the TLP-AA-Kit has potential for application as a useful tool for the detection of severe periodontitis in population-based surveillance. Further large-scale studies are needed to validate the current study’s results and to evaluate the potential of the TLP-AA-Kit as a large-scale, cost-effective surveillance strategy for periodontitis and associated systemic conditions. Although the scoring method of the TLP-AA-Kit may need to be refined to detect cases of early disease, the speed of the test will enable its broad application and provide critical health status information to medical professionals.

## Figures and Tables

**Table 1:** Characteristics of the study population according to periodontitis classification.

	Total	Periodontitis				
		No	Mild	Moderate	Severe	
	*n* = 105	*n* = 72	*n* = 11	*n* = 17	*n* = 5	*p* *
*n* of teeth, median (IQR)	28 (28–28)	28 (28–28)	28 (28–28)	28 (27–28)	28 (25–28)	0.200
Average PPD (mm), median (IQR)	1.9 (1.7–2.1)	1.8 (1.7–1.9) ^a^	2.1 (1.9–2.2) ^b^	2.1 (2.0–2.3) ^b,c^	2.5 (2.3–2.6) ^c^	<0.001
Average CAL (mm), median (IQR)	1.9 (1.7–2.1)	1.8 (1.7–1.9) ^a^	2.0 (1.9–2.1) ^b^	2.2 (2.1–2.3) ^b,c^	2.6 (2.4–2.7) ^c^	<0.001
BOP (%), median (IQR)	7.1 (4.8–15.5)	6.5 (3.3–10.7) ^a^	12.5 (6.5–22.0) ^b^	14.9 (4.8–22.0) ^b^	19.6 (8.3–41.7) ^b^	0.029
Denture use, *n* (%)	2 (1.9%)	2 (2.8%)	0 (0%)	0 (0%)	0 (0%)	0.820
Presence of dental plaque, *n* (%)						0.470
None	43 (41.0%)	33 (45.8%)	5 (45.5%)	3 (17.6%)	2 (40.0%)	
Plaque on gingival margins only	42 (40.0%)	27 (37.5%)	3 (27.3%)	10 (58.8%)	2 (40.0%)	
Plaque also elsewhere	20 (19.0%)	12 (16.7%)	3 (27.3%)	4 (23.5%)	1 (20.0%)	
TCI (%), median (IQR)	50.0 (38.9–50.0)	50.0 (38.9–50.0)	50.0 (38.9–50.0)	50.0 (38.9–50.0)	50.0 (50.0–55.6)	0.710
Tooth brushing ≥2 times/day, *n* (%)	89 (84.8%)	58 (80.6%)	11 (100%)	16 (94.1%)	4 (80.0%)	0.240
Interdental cleaning devices use, *n* (%)	58 (55.2%)	41 (56.9%)	7 (63.6%)	7 (41.2%)	3 (60.0%)	0.610
Regular dental check-ups, *n* (%)	21 (20.0%)	14 (19.4%)	5 (45.5%)	1 (5.9%)	1 (20.0%)	0.086
Age, mean (s.d.)	40.1 (12.5)	37.3 (11.8) ^a^	39.3 (14.0) ^a,b^	47.7 (8.0) ^b,c^	56.6 (9.4) ^c^	<0.001
Sex, *n* (%)						
Men	71 (67.6%)	46 (63.9%)	7 (63.6%)	14 (82.4%)	4 (80.0%)	0.460
Women	34 (32.4%)	26 (36.1%)	4 (36.4%)	3 (17.6%)	1 (20.0%)	
High-risk drinking, *n* (%)	20 (19.0%)	14 (19.4%)	0 (0%)	5 (29.4%)	1 (20.0%)	0.290
Current smoker, *n* (%)	7 (6.7%)	6 (8.3%)	0 (0%)	1 (5.9%)	0 (0%)	0.690
Habitual exercise, *n* (%)	37 (35.2%)	25 (34.7%)	6 (54.5%)	3 (17.6%)	3 (60.0%)	0.140
Poor sleep, *n* (%)	33 (31.4%)	25 (34.7%)	1 (9.1%)	5 (29.4%)	2 (40.0%)	0.370
Obesity, *n* (%)	12 (11.4%)	6 (8.3%)	0 (0%)	5 (29.4%)	1 (20.0%)	0.048
Diabetes, *n* (%)	17 (16.2%)	8 (11.1%)	2 (18.2%)	7 (41.2%)	0 (0%)	0.017

BMI = body mass index, BOP = bleeding on probing, CAL = clinical attachment loss, IQR = interquartile range, PPD = probing pocket depth, s.d. = standard deviation, TCI = tongue-coating index. * *p* value for the comparison among groups. Different letters; a, b, and c, indicate statistically significant differences between groups. Underlined text indicates data with significant adjusted standardized residuals.

**Table 2:** TLP-AA-Kit scores according to the selected behavior prior to examination.

		Yes		No		*p* *
Antibiotic usage within a month	*n* (%)	15	(14.3)	90	(85.7)	
	TLP-AA-Kit score, median (IQR)	1.0	(1.0–2.0)	1.0	(1.0–1.5)	0.865
Tooth brushing within an hour	*n* (%)	43	(41)	62	(59.1)	
	TLP-AA-Kit score, median (IQR)	1.0	(1.0–1.5)	1.0	(1.0–1.5)	0.200
Tongue cleaning within an hour	*n* (%)	9	(8.6)	96	(91.4)	
	TLP-AA-Kit score, median (IQR)	1.0	(1.0–1.0)	1.0	(1.0–1.5)	0.360
Mouth rinse usage within an hour	*n* (%)	6	(5.7)	99	(94.3)	
	TLP-AA-Kit score, median (IQR)	1.25	(1.0–1.5)	1.0	(1.0–1.5)	0.548
Taken a meal within an hour	*n* (%)	74	(70.5)	31	(29.5)	
	TLP-AA-Kit score, median (IQR)	1.0	(1.0–1.5)	1.0	(1.0–2.0)	0.227
Smoking within an hour	*n* (%)	5	(4.8)	100	(95.2)	
	TLP-AA-Kit score, median (IQR)	1.5	(1.0–1.5)	1.0	(1.0–1.5)	0.320

IQR = interquartile range. * *p* value for the comparison between groups.

**Table 3:** Correlation between TLP-AA-Kit score and periodontal parameters.

Parameter	ρ	*p*
Average PPD	0.224	0.022
Average CAL	0.235	0.016
BOP	0.164	0.095

ρ = Spearman’s rank correlation coefficient, BOP = bleeding on probing, CAL = clinical attachment loss, PPD = probing pocket depth.

**Table 4:** Sensitivity, specificity, correct classification, and positive and negative likelihood ratios of the TLP-AA-Kit score for periodontitis cases.

**Moderate/Severe Periodontitis**					
**TLP-AA-Kit Score Value**	**Sensitivity**	**Specificity**	**Classification**	**LR+**	**LR−**
≥1	100.0%	0%	21.0%	1.00	
≥1.5	59.1%	68.7%	66.7%	1.89	0.60
≥2	54.6%	91.6%	83.8%	6.47	0.50
≥2.5	31.8%	95.2%	81.9%	6.60	0.72
≥3	22.7%	98.8%	82.9%	18.86	0.78
≥3.5	4.6%	100%	80.0%		0.95
≥4	0%	100%	79.1%		1.00
**Severe Periodontitis**					
**TLP-AA-Kit Score Value**	**Sensitivity**	**Specificity**	**Classification**	**LR+**	**LR−**
≥1	100%	0%	4.8%	1.00	
≥1.5	100%	66.0%	67.6%	2.94	0
≥2	100%	86.0%	86.7%	7.14	0
≥2.5	60.0%	92.0%	90.5%	7.50	0.43
≥3	40.0%	96.0%	93.3%	10.00	0.63
≥3.5	0%	99.0%	94.3%	0	1.01
≥4	0%	100%	95.2%		1.00

LR+ = positive likelihood ratio, LR− = negative likelihood ratio.

## Data Availability

The data presented in this study are available upon reasonable request.

## Supplementary Materials

The following are available online at https://www.mdpi.com/2304-6767/8/3/98/s1, Figure S1: Receiver operating characteristic curve of the TLP-AA-Kit score for severe periodontitis among participants without a smoking habit, obesity, or diabetes, Table S1: Sensitivity, specificity, correct classification, and positive and negative likelihood ratios of the TLP-AA-Kit score for severe periodontitis among participants without a smoking habit, obesity, or diabetes (*n* = 73).
