# Supplementary material for: A Preliminary Study on the Ability of the Trypsin-Like Peptidase Activity Assay Kit to Detect Periodontitis
Source: Dent J (Basel). 2020 Sep 1;8(3):98. doi: 10.3390/dj8030098 (PMC7558553; doi:10.3390/dj8030098)
Supplement: Supplementary file 1 [file dentistry-08-00098-s001.pdf]

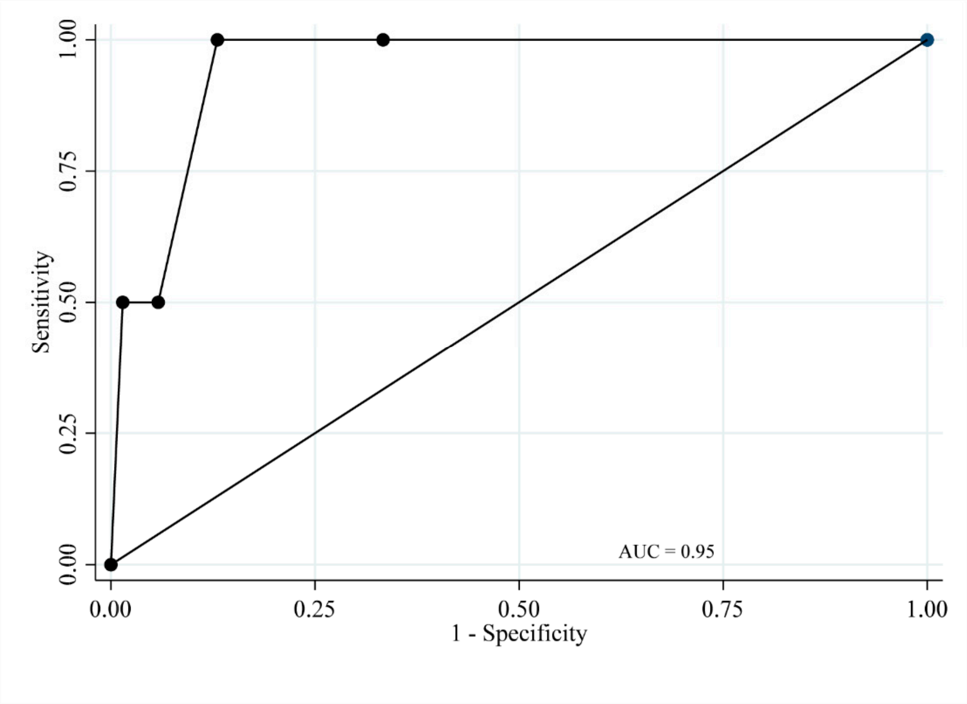

Figure S1. Receiver operating characteristic curve of the Trypsin-Like Peptidase Activity Assay Kit (TLP-AA-Kit) score for severe periodontitis among participants without a smoking habit, obesity, or diabetes.

Table 1. Sensitivity, specificity, correct classification, and positive and negative likelihood ratios of the TLP-AA-Kit score for severe periodontitis among participants without a smoking habit, obesity, or diabetes (*n* = 73).

TLP-AA-Kit Score Value	Sensitivity	Specificity	Classification	LR+	LR-
≥ 1	100%	0%	5.5%	1.00	
≥ 1.5	100%	66.7%	68.5%	3.00	0
≥ 2	100%	87.0%	87.7%	7.67	0
≥ 2.5	50.0%	94.2%	91.8%	8.63	0.53
≥ 3	50.0%	98.6%	95.9%	34.50	0.51
> 3	0%	100%	94.5%		1.00

LR+ = positive likelihood ratio, LR- = negative likelihood ratio
